# Probabilistic and rich individual working memories revealed by a betting game

**DOI:** 10.1038/s41598-023-48242-x

**Published:** 2023-11-27

**Authors:** Syaheed B. Jabar, Kartik K. Sreenivasan, Stergiani Lentzou, Anish Kanabar, Timothy F. Brady, Daryl Fougnie

**Affiliations:** 1 Program in Psychology, New York University Abu Dhabi, PO Box 129188, Saadiyat Island, Abu Dhabi, United Arab Emirates; 2 Program in Biology, New York University Abu Dhabi, PO Box 129188, Saadiyat Island, Abu Dhabi, United Arab Emirates; 3 Center for Brain & Health, New York University Abu Dhabi, PO Box 129188, Saadiyat Island, Abu Dhabi, United Arab Emirates; 4 Department of Psychiatry, Massachusetts General Hospital, Boston, USA; 5 Department of Psychology, University of California, San Diego, La Jolla, USA

**Keywords:** Human behaviour, Working memory

## Abstract

When asked to remember a color, do people remember a point estimate (e.g., a particular shade of red), a point estimate plus an uncertainty estimate, or are memory representations rich probabilistic distributions over feature space? We asked participants to report the color of a circle held in working memory. Rather than collecting a single report per trial, we had participants place multiple bets to create trialwise uncertainty distributions. Bet dispersion correlated with performance, indicating that internal uncertainty guided bet placement. While the first bet was on average the most precisely placed, the later bets systematically shifted the distribution closer to the target, resulting in asymmetrical distributions about the first bet. This resulted in memory performance improvements when averaging across bets, and overall suggests that memory representations contain more information than can be conveyed by a single response. The later bets contained target information even when the first response would generally be classified as a guess or report of an incorrect item, suggesting that such failures are not all-or-none. This paradigm provides multiple pieces of evidence that memory representations are rich and probabilistic. Crucially, standard discrete response paradigms underestimate the amount of information in memory representations.

## Introduction

Working memory (WM) refers to the capacity to keep information active and accessible when it is no longer present in the environment. This capacity is involved in nearly all domains of cognition and its central importance is reflected by its high correlation with measures such as fluid intelligence^1,2^ and academic success^3,4^. Despite the importance of WM in our daily lives, it has a surprisingly limited capacity^5–8^. The amount of information that can be stored online in working memory (WM) is of central theoretical and practical importance in understanding the limits of our cognitive abilities. Importantly, memory for simple features is correlated with memory for complex objects^9,10^, enabling insights into everyday limitations in working memory through the study of memory for simple visual features which can be presented in a well-controlled manner.

Contemporary theories have constructed elaborate models of WM based on data from adjustment tasks, which require participants to report the color of a remembered object from a color space. Researchers perform many trials of such studies to generate aggregate distributions in order to differentiate between models of WM^5,11–14^. While this approach has yielded many important insights into memory, it compresses knowledge of individual memories into point estimates. This is suboptimal, as the richness of individual memories is a core issue at stake in theories of memory representation.

There are several theoretical perspectives on the richness of individual memories. Some argue that most of the sensory information is lost by the time a decision must be made^15^ such that memories consist of a point representation only (e.g., a particular shade of red) rather than a full sensory distribution. Models of visual working memory where memories ‘drift’ over time^16^ or with noiseless representations^17^ often assume this is true. Another possibility is that memories may be ‘rich’—by rich we mean a representation that is more than a point estimate, but also includes a sense of certainty, such as “pink but I’m not sure”. Consistent with this, researchers have shown that subjective measures of confidence^18–20^, or other measures that tap into a sense of certainty^11,21^, correlate with performance on a per trial basis. Since working memory is for more than just passively encoding objects, but is for reasoning and acting on information, rich representations would be beneficial as it would allow one to act with more or less confidence depending on the uncertainty of the information^21^. Yet another possibility is that memory representations are not only ‘rich’, but also ‘probabilistic’, consisting, for example, of stored distributions over a feature space or a full population code^6,22–25^ or a large number of likely samples^26^. Note that our usage of probabilistic could reflect a continuum—if a representation consisted of a handful of samples^26^ the resulting memory representation would have some distributional properties, without being a full probability distribution.

**Figure 1:**
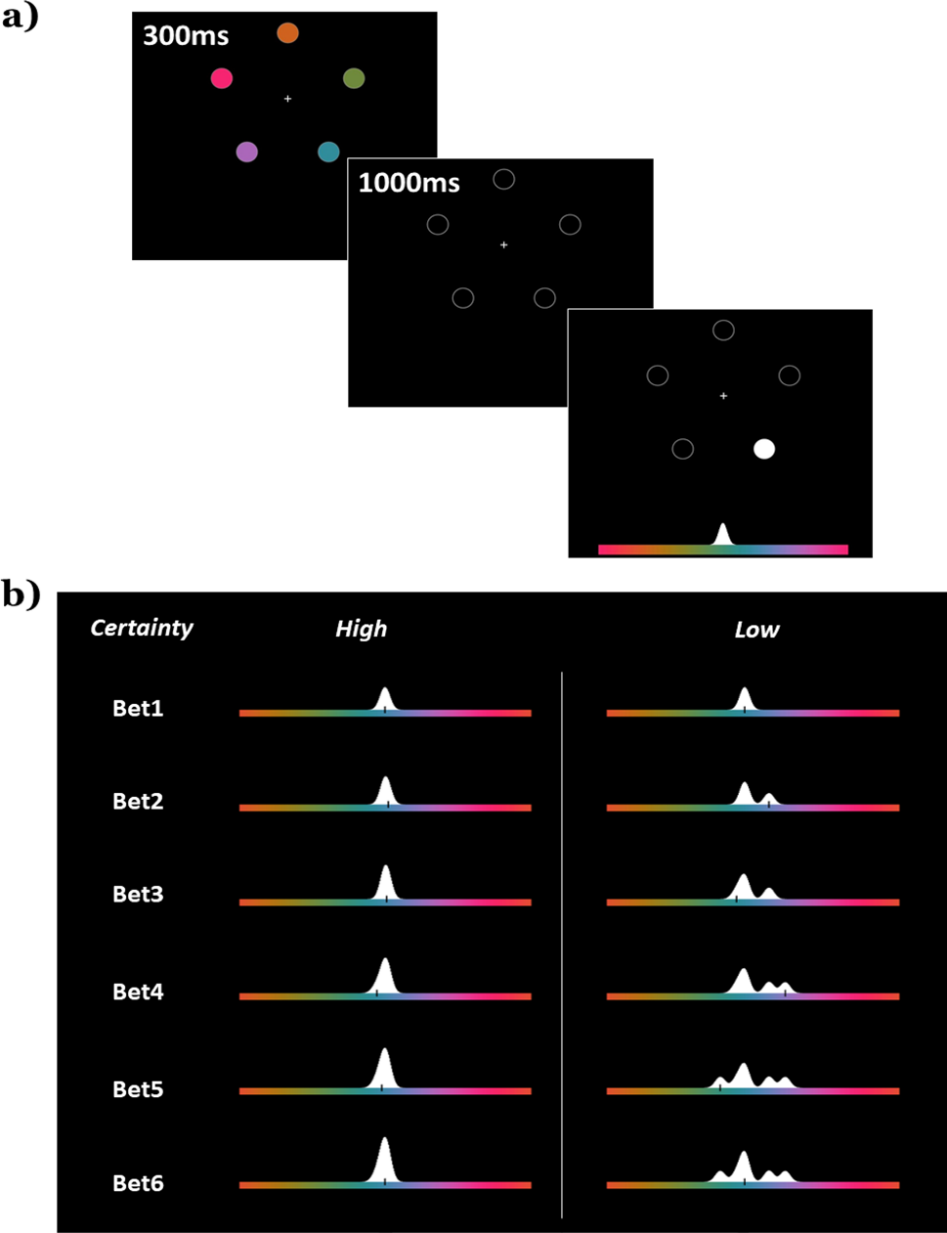
Experiment protocol. (**a**) Five color targets were shown, followed by a delay period. A color response bar then appeared, and one of the target locations was highlighted. In Experiment 1 participants were always asked to make 6 ‘bets’. (**b**) In the first bet, participants used a mouse to laterally shift the colors of the response bar. Participants were asked to place the color they remembered in the cued location at the center of the bar, where the white Gaussian is. Participants confirmed their choice with a mouse click. For the 2nd to 6th bets, another Gaussian (with half the height of the original), appeared at a random position on the bar, and participants were asked to move the mouse to place the Gaussian over what they thought was the color they remembered, again confirming the bet with a mouse click. This was repeated for six bets per trial. Participants were free to spread out the bets as they wished. Changes to the profile were previewed to participants before each response. If they were very certain, they could stack the bets, forming a narrow uncertainty profile (left column). Doing this could earn them more points if they were on-target, but at the risk of no reward if there was no height of the drawing over the target color. In this case, participants would have received 132 points. If uncertain (right column), participants could spread the bet, as a lower-risk, but lower-reward option (in this case receiving 21 points due to the reduced height at the target color).

If WM representations might be rich and probabilistic, then why are our report methods discrete and limited? Research has assumed that discrete reports are a reasonable summary of internal information, even if that information is complex (e.g., the mean of a probability distribution). However, it could also be that representations are sufficiently complex to preclude description by discrete responses. Alternatively, responses may be sub-optimal relative to internal knowledge. If either of these are true, then it is incomplete and potentially misleading to assume that discrete responses are equivalent to the memory. For example, if responses are noisy samples from a probability distribution^26–28^, then existing estimates of memory constraints could be significantly underestimating the amount of information represented in memory. Some models predict rich potentially asymmetric internal distributions in feature space^24,25,29^ in which there is no straightforward conversion of the probability distribution to a single response. To determine the richness of WM representations, we developed a betting game task (Fig. 1) that encouraged participants to report probability distributions of individual memory representations (*uncertainty profiles*) by constructing a distribution over color space rather than a single report plus uncertainty. The richness of reports allowed us to compare how the reported uncertainty profile compared to the first report, to test whether discrete reports are representative of stored information. The critical question is whether taking in the full information from reports (for example taking the average of multiple responses) reveals more information than a single report. This could occur if representations are probabilistic, and responses are either noisy samples or sub-optimal summaries of stored probabilistic information. In this case, additional responses can, on average, yield additional information (i.e., be biased towards the true value). However, if representations are point estimates (or if discrete responses can fully capture complex probabilistic information) then additional responses will yield *no* new information and will *not* improve performance. Critically, a benefit from multiple responses requires an *asymmetry* in future bets distributed around the first response that is biased towards the true value. Therefore, content-independent notions of confidence^30^ or noisy responses should provide *no* benefit as they contain no inherent bias towards the true value. Thus, gathering multiple responses is a method of determining whether existing tasks fully capture the information contained within participants working memory representations. Refer to the “Supplemental Materials” for different models and the predictions these models would make about multiple reports.

To preview the results, participants created uncertainty profiles that were close facsimiles to the error distribution across trials. Moreover, the placement of bets tracked internal uncertainty—when bets were more widely spaced, participants were less accurate, and vice versa, suggesting some access to the underlying uncertainty in a memory representation. Most provocatively, participants had more information about the memory items than was contained in the first report, regardless of whether participants were approximately on-target or showed guess-like error magnitudes. Later bets also biased participants’ uncertainty profiles towards the target, creating asymmetric distributions that cannot be explained if memory representations are only discrete. These findings suggest that our memories are complex probability distributions, but that we are unable to fully capture this information with single reports. Existing theories/models—built on assumptions that equate responses with internal representations—thus underestimate the amount of information in memory representations.

## Methods

### Experiment 1

#### Participants

Forty naïve participants were recruited for Experiment 1. Demographic (14 female, 26 male, age range 18–32 [median = 23.5]) information were obtained from self-reports (of age, gender, handedness) collected before the main experiment (this is also true for subsequent experiments). This sample size is based on pilot data collected in-lab. With twenty participants we had sufficient power to detect the decrease in cumulative error about 80% of the time. Since we conducted this study online and we expected increased noise (e.g., due to variations in participant equipment set-up), we decided to double the sample size. All participants declared normal or corrected-to-normal visual acuity and color vision. Participants were recruited online (via http://www.prolific.co) for a base allowance of 5.20 GBP per hour and were told that they could also receive a monetary bonus depending on their performance (mean bonus = 2.00 GBP). The experiments were approved by the New York University Abu Dhabi Institutional Review Board and carried out in accordance with relevant guidelines and regulations. Informed consent was obtained from all participants. As six participants had abnormally large guess rates (> 66%) and were either non-compliant (randomly placing bets) or could not perform the task, we removed them from the analyses. This cutoff was used for all subsequent experiments as well.

#### Apparatus and stimuli

Because of the online nature of the experiment, stimulus sizes could vary slightly depending on the participant’s screen size/viewing distance. Assuming that a 27-inch 1920 × 1080 resolution monitor placed 70 cm away was used, targets would be located approximately 4° away from the center of the screen, with the target circles themselves occupying 1° of visual angle. The background of the screen was black throughout the experiment. Targets appeared in the same five equidistant locations (see Fig. 1a for positions). The response color bar was approximately 6° from the center of the screen with a thickness of 1.0°. Effort was made particularly to ensure that the drawn display fit within the participant’s browser page. Participants were also asked to maximize their browser window prior to launching the experiment. The colors chosen for a given trial and for the response bar were sampled from the MemToolbox^31^ 360° color space, translated into RGB assuming an equal energy whitepoint. All experiments were programmed entirely in HTML Canvas/JavaScript. Data obtained from this study and a demo HTML is available online: https://osf.io/7srv4/. This study was not preregistered.

#### Procedure

In Experiment 1, each trial began with a 500 ms blank screen with a fixation cross. Five colored stimuli appeared on-screen for 300 ms. These five colors were randomly chosen for each trial. Stimulus presentation was followed by another blank screen for 1000 ms, during which time participants maintained the color information in WM. After this delay period, the location of one of the five targets (each location equally probable) was then cued as the tested location. The response color bar appeared and for the first bet, participants used a mouse to laterally shift the colors of the response bar (see Fig. 1a). Participants were asked to make the color they thought was in the cued location to be the center of the bar, where a white Gaussian distribution was located (standard deviation of 6° in color space). Participants confirmed their choice with a mouse click.

For the 2nd to 6th bets, a standard Gaussian appeared at a random position on the bar, and participants were asked to move the mouse to place the Gaussian over the color bar, again confirming the bet with a mouse click (iteratively, for a total of six bets). With these additional bets, participants were told to spread them according to how certain they were, and that simply stacking the bets on top of each other would reduce the height of the distribution built. Participants were also explicitly told that the points they earned per trial was dependent on the height of their drawn distribution at the correct color. During each bet, the updated uncertainty profile (based on current mouse position) was updated per frame and previewed to participants. The six Gaussians summed to create a final uncertainty profile for that trial. The choice in shape of individual bets is not meant to imply a similar shape in the internal error distributions; the use of Gaussians allows participants to make graded and non-discrete responses over the feature space. Participants were free to stack responses on top of one another (see Fig. 1b, left column) or to spread it across a larger color space (right column). As a result, the uncertainty profile ‘drawn’ at the end of the sixth response need not have a Gaussian shape, and can be asymmetrical. Note that the Gaussians appeared in a random location between responses to prevent stereotyped successive clicks (e.g., participants responding in a lazy manner) on the same color location, and participants had to wait a minimum of 300 ms and make a lateral mouse movement before they could register the next click.

To encourage participants to report something resembling the true internal uncertainty, participants were awarded points based on the height of the final drawn distribution over the target color:

$$ {\text{points}} = {\text{Height}}\;{\text{of}}\;{\text{final}}\;{\text{distribution}}\;{\text{at}}\;{\text{target}}\;{\text{color}}*{5}00 $$

The *first* bet was twice as tall (worth twice as many points) to encourage participants to place the first bet accurately. Even if memory representations have uncertainty, the optimal strategy when placing bets would be to stack all bets on the peak of their internal uncertainty distribution. We implemented subtle diminishing returns to rewarded points when stacking bets on existing bets. Critically, while this might encourage participants to spread bets (although pilot data suggests it does not impact participants performance) it would not lead to *meaningful* bet placement unless the participant already had information about an item not captured in the first response. This penalty was scaled by height. Specifically, the height of the Gaussian component to be added was penalized by the current height of the drawn profile raised to a penalty parameter. For example, if the current bet is *b*, and the profile already drawn due to the previous bets is *y*, then the new bet *y*′ is:

$$ y^{\prime} \, = y + {\text{standard}}\;{\text{Gauss}}*({1} - {\text{Height}}_{{y\;{\text{at}}\;b}}^{{0.{4}}} ),\;{\text{where}}\;0.{4}\;{\text{is}}\;{\text{the}}\;{\text{penalty}}\;{\text{value}} $$

The taller the current height at the new bet value, the smaller the possible gain in height (and therefore the smaller the gain in points). Importantly, this penalty was built into the visualization of the drawn distribution seen by participants. After the six bets were placed, the trial score, total score, as well as the current accumulated monetary bonus (every 200 points earned 0.10 GBP) were displayed on the feedback screen until the participant re-centered the mouse on the central fixation. To be clear, the theoretical maximum number of possible points per trial, despite the penalties to stacking, still occurs if the participant places their all their bets on the target color. This maximum possible points per trial was 146. Each participant first completed 6 practice trials, followed by 150 main trials, with a break every 50 trials. Practice trials were excluded from the analyses. The primary reason for the penalty is that an optimal observer would stack *all* bets on the same point, regardless of whether they were certain or uncertain. Although it is unknown whether this penalty encourages or is required for spreading bets in actual data, it is important to clarify that spreading bets, by itself, is insufficient to cause our observed results.

### Motor control experiment

A control study of Experiment 1 was done on 20 additional naïve participants (10 female, 10 male, age range 18–31 [median = 23.5]). We used the same betting protocol as Experiment 1 but stripped away the memory demands by leaving the stimulus on screen during the delay and report in order to isolate the response error (and other non-memory sources of error) involved in reporting an onscreen stimulus. Because precision was expected to be high (and therefore data would be low in noise), participants were only required to do 80 trials, preceded by 6 practice trials. Three participants were removed due to random betting, and the remaining participants had a very small magnitude of error (first bet mean absolute error = 3.91°) showing that non-memory sources of error are low.

### Experiment 2

As a replication of Experiment 1, and to minimize the possibility that the predictability of always having to make multiple responses affected the precision of the first bet, Experiment 2 was conducted using 40 additional naïve participants (11 female, 29 male, age range 18–31 [median = 23]). We used the same betting protocol as Experiment 1 except that we randomly interspersed trials that required only a single response (e.g., just the first bet) amongst the trials that required placing a sequence of 6 bets. Participants completed 100 trials of each type. Critically, the response condition was not known until the second response; thus, participants could not anticipate at the time of the first response whether they would have the opportunity to make multiple bets. Of the 40 participants, 9 made many random clicks (guess rate > 66%, same cutoff as Experiment 1) and were dropped from the analysis.

## Results

### Evidence that the reported distributions incorporate uncertainty

To explore the degree to which participants’ bets captured their memory uncertainty, we first looked at whether the spread of bets predicted memory performance. This analysis leverages the findings that memories vary in quality^11,12^ and that participants have some metaknowledge of the uncertainty in memory^18,21,32,33^. Thus, to maximize performance, a participant should place bets narrowly when the uncertainty of the true color is low and spread bets widely when uncertainty is high. Indeed, we found significant positive correlations (*p* < 0.05) between the magnitude of the error of the first response and the median absolute distance between adjacent bets for 32 of the 34 participants (mean* r* = 0.393). These individual correlations were Fisher-transformed (mean *z* = 0.430, *95% CI* = [0.010, 0.977]) and a *t*-test found this distribution to be significantly different from zero (*t*(33) = 12.57, *p* < 0.001). Similar results were found using other measures of the bet spread, including the standard deviation (mean *z* = 0.379) or the interquartile range^34^ (mean *z* = 0.362) of the uncertainty profile.

### Evidence that uncertainty profiles reflect trial-specific information

**Figure 2:**
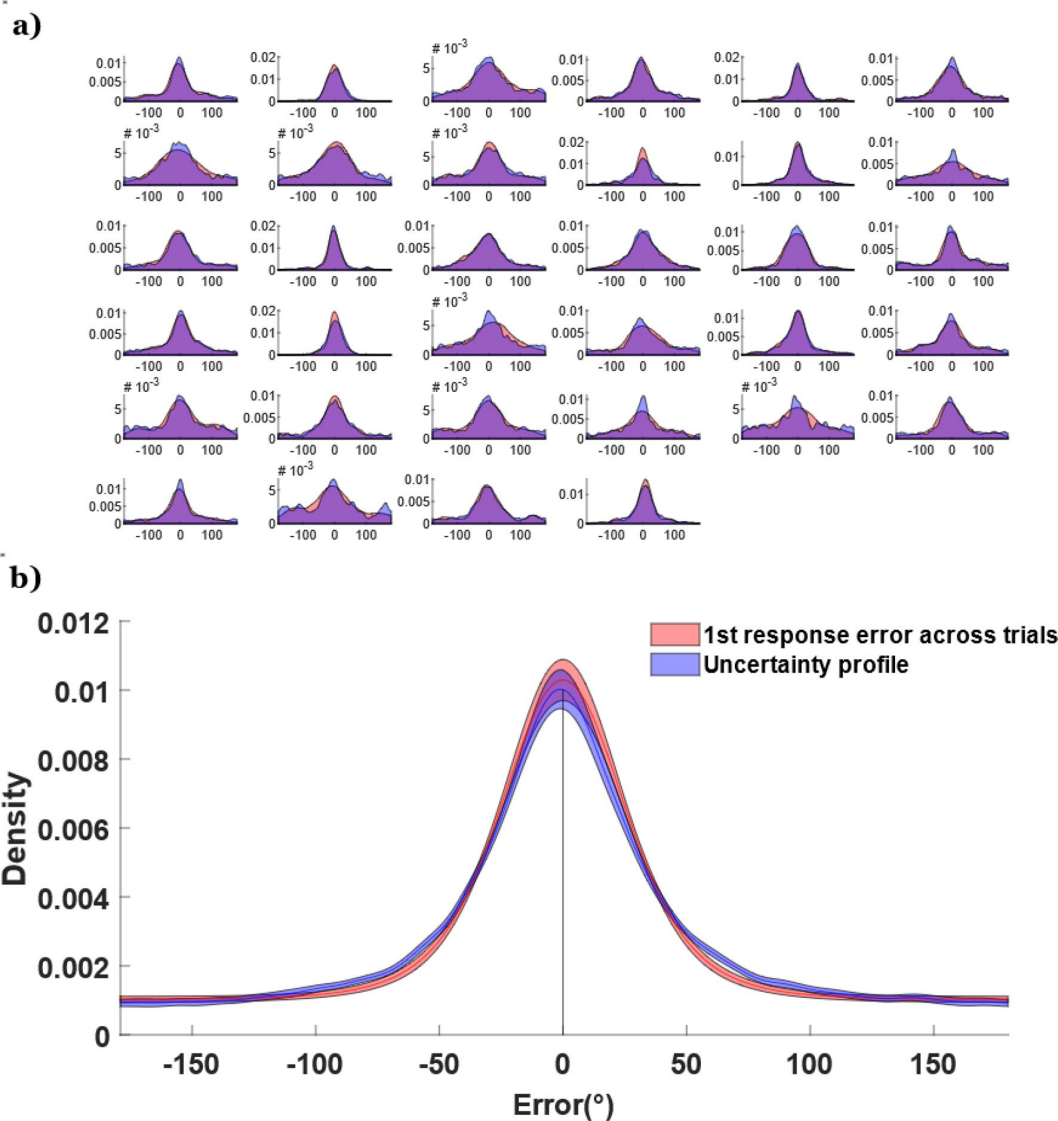
Comparing the error distribution of the first response across trials (red) versus the uncertainty profiles (after all six bets) averaged across trials (blue). (**a**) The comparison between the two distributions for each of the 34 participants. (**b**) Aggregate of the distributions across participants. The shaded region represents 1 standard error above and below the mean.

The above analysis demonstrates that the way the bets are placed reflects the error in participants’ first response, but how closely does the reported uncertainty within trials match participants’ across-trial error, as assessed by the profile of the error in first responses across trials? If participants were accurately recreating the uncertainty in memory and sampling from this to derive first responses^27^, the across trial error distributions would reflect the average of the uncertainty profiles reported within a single trial (e.g., if a participant has high uncertainty this individual should distribute bets more widely). We examined this possibility using two-sample Kolmogorov–Smirnov (*KS*) tests^35^ to compare the across-trial error distribution (error of the first response relative to the correct answer) to the trial averaged uncertainty profile for each participant. Uncertainty profiles were circularly shifted to align the target colors and averaged at each integer value of the color space (e.g., at 360 discrete points). For 31 of the 34 participants, the *D*-value (the *KS* statistic which measures the maximum difference between the two empirical cumulative distribution functions) was small (mean *D* = 0.042, *SD* = 0.015) and non-significant (*p* > 0.05), highlighting that the average reported uncertainty was not significantly different from the across-trial error of the first response (Fig. 2). To construct a bootstrap simulation to see the distribution of *D*-values under the null hypothesis we compared the first bet error distributions and uncertainty profiles from non-paired, random participants, rather than for paired individuals. These random pairings were repeated 1000 times to yield a 95% confidence interval [0.088 0.124] for the *D* statistic under the null hypothesis (that the two distributions do not share the same shape). Because the actual *D* statistic fell below this range, it suggests that the distribution of bets is being informed by the participants underlying memory uncertainty.

### Evidence that uncertainty profiles are asymmetric

One strategy that participants could use would be to spread bets around a best-remembered color according to a notion of confidence, which would produce roughly equal numbers of bets on each side of the first response. However, bet placement was more asymmetric than this strategy would predict: 65.7% (*SD* = 3.1%) of the distribution was on the side with more bets. This is larger than would be expected by chance (even due to the limited number of responses). To show this, we compared this amount to a control analysis in which bets 2–6 were assigned random signs. The resulting asymmetry in bets, 57.1% (*SD* = 4.0%) of the distribution on the side with more bets, was significantly lower than the actual data, *t*(33) = 8.34, *p* < 0.001.

### Evidence that the bets contain more information than found in the first response

Additional responses were not only asymmetric relative to the first response, but this asymmetry was related to the target. This is critical, because models and theories from continuous report tasks estimate memory representations based on a single response, which either explicitly or implicitly assumes that this response is synonymous with the memory. However, such tasks may be underestimating how much is maintained in memory by confusing uncertainty in individual reports with uncertainty in the content of working memory. Does the quality of memory get better if we consider multiple reports?

**Figure 3:**
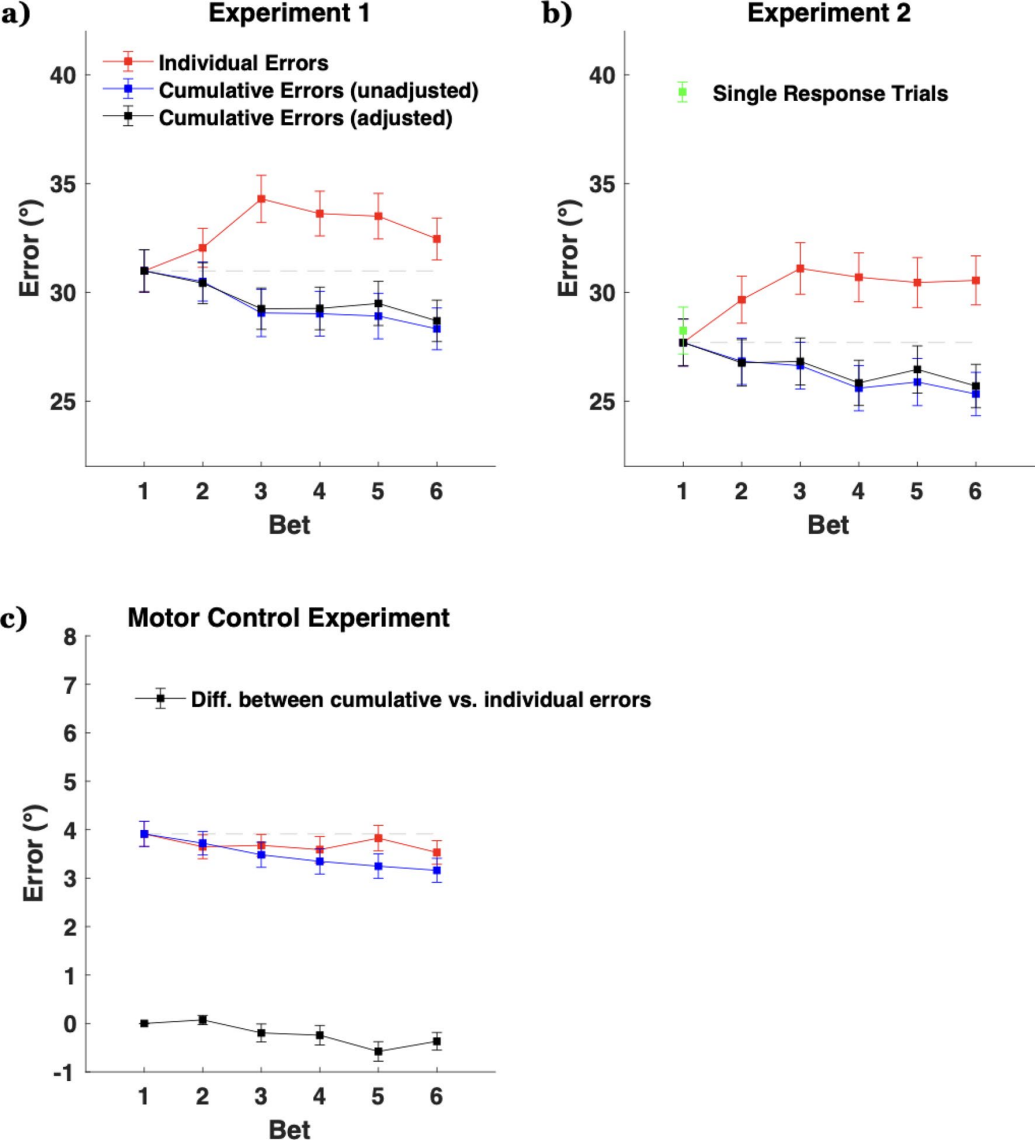
Individual response errors (red) and cumulative errors (blue) as a function of response order. Cumulative errors are calculated as the error of the mean of responses. For example, the cumulative error for response 3 would be influenced by response 2 and response 1, whereas the individual response error for response 3 is solely determined by that bet. (**a**) Errors for Experiment 1. Adjustments were made on the cumulative errors based on the results of the motor control experiment to try to minimize the impact of motor noise. (**b**) The equivalent errors for Experiment 2. The green marker is the mean for the single response trials. (**c**) Errors for the motor control experiment where the stimuli stayed on-screen.

To investigate this, we analyzed the placement of individual bets, as well as the cumulative circular average of the bets (e.g., if the first bet was a color 10° clockwise and the second was 4° counterclockwise, the cumulative average would be 3° clockwise). As shown in Fig. 3, the first bet placement is the most accurate, with a relatively monotonic decline in the accuracy of individual bets across the trial (when considering the center of bets in isolation). To examine this, we subjected the bets to a one-way ANOVA with the six levels of bet order as a within-subjects factor. Errors generally increased with bet number (bet 1 = 31.0°, bet 6 = 32.5°), *F*(5, 165) = 3.39, *p* = 0.006,* η*^2^ = 0.093. This result is not surprising given that the first response was worth double the points of subsequent responses. Since participants are asked to and were given incentive to place their most confident bets first, it stands to reason that subsequent bets would be associated with less confidence and likely have larger error magnitudes, particularly since revisiting the top bet was subtly discouraged. On top of that, the subsequent responses occurred after increasing delays and potential interference from previous responses, adding more noise.

Despite being less accurate than the first responses, subsequent responses could nevertheless contain additional, unique information about the target item^28^ if memory is rich and probabilistic, but not if memory simply consisted of a point representation or a point representation plus a sense of uncertainty. This is something that can be shown computationally (see “Supplemental Materials”). To test this in our data, we examined whether additional bets were providing novel information not contained in the first response by testing whether the center of the uncertainty profile approaches the true color with repeated placement of bets. We subjected mean bet placement to a one-way ANOVA and found that performance improved with additional bets (bet 6 = 28.3°), *F*(5,165) = 5.35, *p* < 0.001,* η*^2^ = 0.140, despite the fact that average error trended worse for the later bets, suggesting that the combination of responses contains more information than any individual response, including the first response. In addition, a further analysis demonstrated that relative to bet 1, bet 2 was made *towards* the direction of the target at a proportion significantly above chance (*M* = 57.7% vs. 50.0%), *t*(33) = 7.47, *p* < 0.001, *BF* = 945,352). The same held true when also considering bets 3 to 6 (*M* = 56.9% vs. 50%), *t*(33) = 8.06, *p* < 0.001, *BF* = 4,415,101. These findings go beyond previous evidence that participants have a notion of memory confidence and imply that *participants have more information in mind than is conveyed by a single report*.

One possibility is that the benefit of averaging is solely due to averaging out individual noisy motor responses or output error (but see Sutterer, Rosca and Woodman^36^ for evidence that motor noise plays no significant role in continuous report tasks). However, motor error is a tiny fraction of the error variance for remembering 5 items and is unlikely to play a decisive role in our effect. To test this directly, we conducted a motor control task (see “Methods”). Unsurprisingly, we observed a benefit when combining multiple responses, *F*(5, 80) = 15.6, *p* < 0.001,* η*^2^ = 0.0.49, but the change in error was less than a degree (bet 1 vs all 6 combined: difference = 0.75°). To show that the benefit of averaging across bets in Experiment 1 is not solely from motor area we added the average amount of cumulative improvement found in the motor control Experiment (black line in Fig. 3c) for responses 2–6 to the corresponding cumulative response error (blue line Fig. 3a) (on a per participant basis). This had the effect of slightly narrowing the measured improvement, but we still found evidence of significantly reduced error when averaging bets, *F*(5, 165) = 3.63, *p* = 0.004,* η*^2^ = 0.099. Motor error cannot explain the effect seen in Experiment 1. Critically, our conclusions are not affected by differences in participant count or subject number. The increase in cumulative precision for Experiment 1 (Bet 1 mean = 31.1°, Bet 6 cumulative mean = 28.3°) was evident within just the first 80 trials, *F*(5, 165) = 4.45, *p* < 0.001,* η*^2^ = 0.119 (this suggests that learning or other changes in behavior over time are not necessary for the findings). Further, sub-sampling down to an equivalent 20 participants still yielded significant results on the cumulative benefit of multiple responses in greater than 99% of resamples.

### Performance for our first response was comparable to single report tasks

An important implication of our results is that estimates of working memory capacity may be based more on *memory responses* than *memory content*. Notably, first response performance is comparable to results of studies that rely on single reports, providing evidence that our findings have implications for the way working memory is performance typically assessed. This is most clearly seen when we decompose our errors using mixture models to facilitate comparisons to previous work (using MemToolBox^31^). We find a guess rate of 37.1% with a set size of five, which is between the 16% found with set size 3 and 59% found with set size 6 in Zhang and Luck^8^. The amount of guessing reflects a capacity of 3.15, compared to a capacity of 2.46 found in Zhang and Luck^8^. Precision *SD* in our online task after factoring out ‘guesses’ was approximately 29.4°, which is also close to our previous (unreported) in-lab iteration of the betting task (with set size of 5, mean guess rate = 31%, *SD* = 28.1°). Since first bet performance is consistent with studies using single reports, this suggests that our paradigm is not underestimating performance due to demand characteristics or low effort. Further, our first bet was weighted higher than others to encourage participants to emphasize this response. In addition, this effect is unlikely one that occurs through learning or strategy (or becoming lazier over time): As stated in the previous section, the increase in cumulative precision was evident within just the first 80 trials. However, to address this issue more fully we ran a replication study that unpredictably interspersed single- and multi-bet trials.

### Interspersing single response trials does not impact the results

We replicated the results under conditions in which participants were uncertain if they were to make one report or multiple bets (Experiment 2). The first bets in Experiment 2 (*M* = 27.6°, *SD* = 12.0°) did not significantly differ in error magnitudes compared to Experiment 1 (*M* = 31.0°, *SD* = 11.3°), *t*(63) = 1.13, *p* = 0.260. Bayes Factor^37^ analyses (*BF* = 0.4) suggest anecdotal evidence for the null hypothesis over the alternative hypothesis (the *BF* approached the cutoff of < 1/3 to support the null but did not surpass it). The single response trial errors (*M* = 28.2°, *SD* = 12.0°) did not differ from the errors on the first bet of the multiple bet trials in error magnitude, *t*(30) = 0.56, *p* = 0.582. For the individual errors of the multiple bet trials, errors increased from bet 1 to bet 6 (*M* = 30.5°, *SD* = 12.6°), with the one-way ANOVA showing *F*(5,150) = 3.45, *p* = 0.006,* η*^2^ = 0.103. For the cumulative errors of the multiple bet trials, errors decreased from bet 1 to bet 6 (*M* = 25.3°, *SD* = 11.1°), with the one-way ANOVA showing *F*(5, 150) = 5.47, *p* < 0.001,* η*^2^ = 0.154. Adjusting these cumulative errors by the motor benefits still suggested a decrease, *F*(5, 150) = 3.70, *p* = 0.004,* η*^2^ = 0.110. Although Experiment 2 tested less participants and used less trials per condition (due to the need to have two conditions) than Experiment 1, our conclusions are affected neither by the differences in participant numbers nor trial numbers between the two studies as demonstrated by our earlier sub-sampling analysis.

Taken together, these results, combined with the fact that our first response accuracy in for Experiments 1 and 2 was in line with previous studies utilizing one response^8^, suggest that multiple responses is not meaningfully distorting results relative to existing approaches. This rules out alternative explanations for our results such as strategic or low-effort first responses.

### Examining speed-accuracy tradeoffs

One concern is that performance improvements reflect that participants sometimes respond lazily on their first response but correct this in later responses. This effect could be expected to be particularly pronounced in Experiment 1, since participants knew they had multiple responses. Of note, in Experiment 2 participants did not know in advance whether they would have the opportunity to make multiple responses. However, we conducted a reaction time (RT) analysis to examine this in more detail. A speed-accuracy tradeoff account predicts that participants sometimes respond quickly instead of accurately. However, there was no correlation between speed of the first bet response (the period between the end of the delay, and the time of the mouse click) and the precision of the response in our studies (*p*s > 0.05). A median split comparing the fastest versus slowest bet 1 responses also yielded a null result (*p*s > 0.05, *BFs* < 1/3) for both experiments. Another prediction of this account is that the change in precision between bet 1 and the mean after the 6 bets had been made would be higher when bet 1 RTs are fastest. However, this ‘improvement metric’ did not correlate with the time taken to make the first bet, and a median split corroborated the null result (all *p*s > 0.05). Note that while we do not report the RT effects for the remaining experiments, we observed similar null effects in these studies. Because guessing or swap errors (responses not sampled from the internal representation of the target) can result in large errors that could affect the pattern of results, we also repeated these speed-accuracy tradeoff analyses with these trials removed, which did not alter the null results. Taken as a whole, these results provide strong evidence that low effort during the first response is not driving our findings, and that additional responses allow for improvement.

### An argument for uncertainty profiles: the case of ‘guess’ and ‘swap’ errors

Thus far we have focused on how the uncertainty profiles provided by participants generally match their aggregate performance, with the cumulative error analyses further suggesting that these profiles contain more information than any single response. However, the benefit of measuring these profiles is that it affords the ability to explore novel issues, like how much information is available about the target when participants’ initial report is far from the target.

Many models of visual WM make claims that distinct ‘states’ underlie some kinds of memory errors. For example, some models suggest that far away responses reflect ‘guesses’, where no target-specific information is available to participants^8^. Other models suggest that some far away reports reflect ‘swaps’, where participants report a non-target item^5,38^ either erroneously/due to cue confusion^38–40^ or strategically^19^. The betting game task allows us to examine whether these responses truly reflect distinct states with no target-specific information by more closely examining the uncertainty profiles for such “swap” trials and “guess” trials.

We classified trials into three trial types: target, swap, and guess trials using the method outlined in Bays, Catalao and Husain^5^ and Schneegans and Bays^38^. Unsurprisingly, based on the first bet errors the majority of trials were classified as target trials (*M* = 73.3%). There were significantly more guess trials (*M* = 20.1%) than swap trials (*M* = 6.6%), (all comparisons, *p* < 0.001). The lack of swap errors likely stemmed from the fact that the locations of the colors were fixed and predictable across trials^5,39^. Only 33 of the 65 available data sets had trials that could be classified as swap trials. To have sufficient power to examine swaps, we combined the data from Experiment 1 and the multiple bet trials from Experiment 2. Swap trials (*M* = 39.1°) did not significantly differ from guess trials (*M* = 43.8°) in terms of bet spread, *t*(26) = 0.88, *p* = 0.387, *BF* = 0.32. When combined with our previous finding that bet spread was correlated with initial response error and thus may reflect memory uncertainty, this result is consistent with other work (e.g., Pratte^19^) demonstrating that confidence ratings do not differ between swap and guess trials. In contrast, the bet spread for the target trials (*M* = 24.2°) was significantly smaller than the two other trial types (*p*s < 0.001). Most importantly, one-way ANOVAs revealed that *all three* trial types had a decrease in cumulative bet errors from bet 1 to bet 6 (*p*s < 0.001,* η*^2^s > 0.01). This suggests that single response methods tend to underestimate the amount of information about the target item that participants store not only for on-target reports, but also for swap or guess reports, and that participants do indeed have target-specific information on such trials. Further, this rules out that the cumulative improvement in bets can arise solely from one source of error (e.g., recovery from swaps).

**Figure 4:**
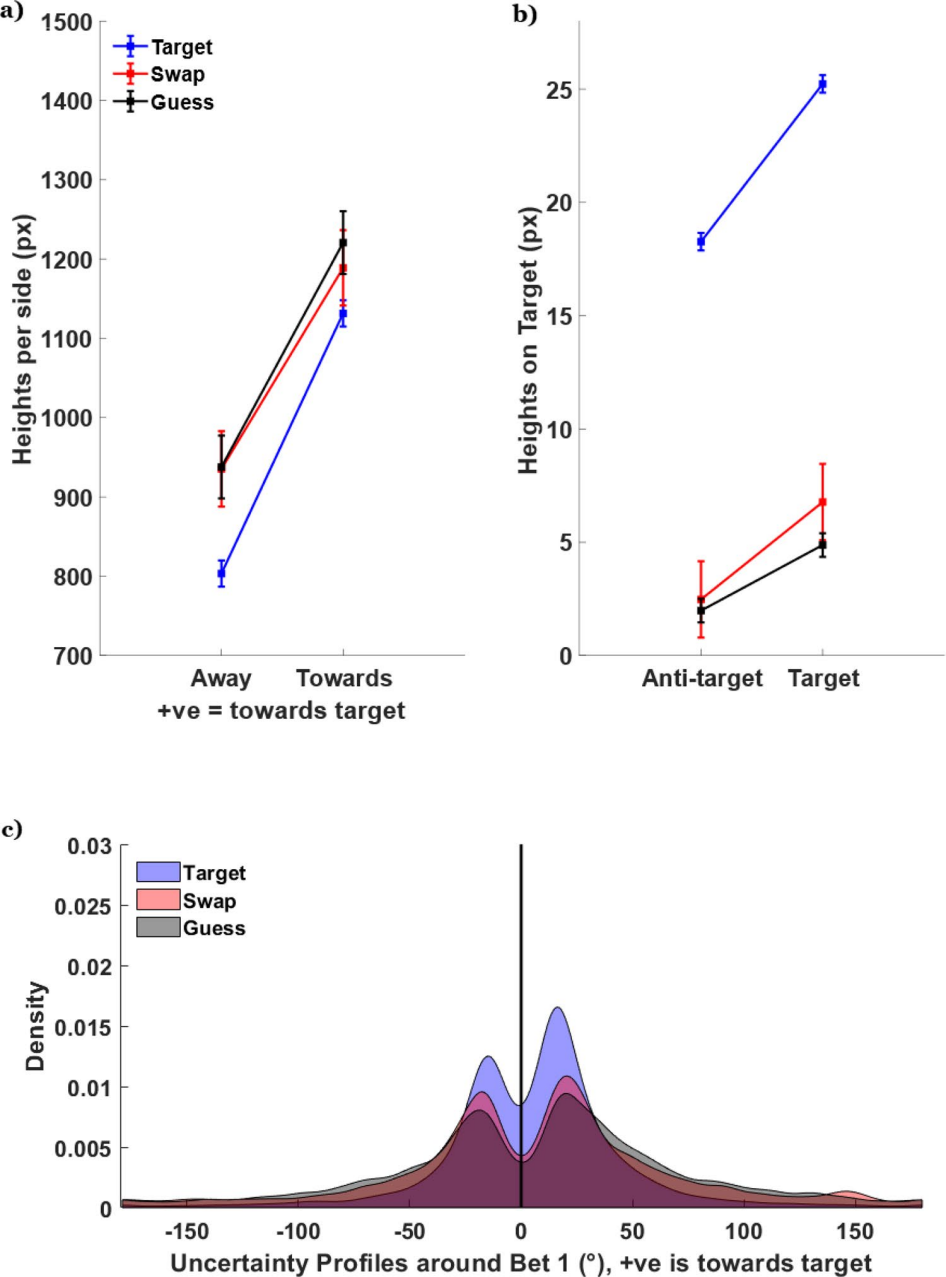
Post-classification analyses. (**a**) Sum of profile heights either towards or away from the target. (**b**) Height over the actual target compared to the anti-target (the color that is equidistant to the target from response 1, but in the opposite direction). (**c**) Distribution of bets around bet 1, with the positive end being towards the true target. Note that the distributions are bimodal because these exclude the Bet 1 contribution and participants tend to spread subsequent bets away from the initial response. Also note that the distribution is asymmetrical, with a greater proportion of the area biased towards the target (+ve x-direction).

Residual target encoding in cases of ‘swap’ and ‘guess’ errors can be further examined via participants’ bet placement over the target color. For example, if a ‘swap’ reflects a complete replacement of the tested item (at either encoding, storage, or retrieval), then we expect the height of the uncertainty profile over the target color to be no higher than over other random positions. To examine this, we took the uncertainty profiles for each trial and centered them on the first response such that errors biased towards the target side were coded as positive (see Fig. 4c). We found that the sum of heights over the estimates biased towards the direction of the target were higher than estimates biased away, for all three target types (Fig. 4a, all *p*s < 0.001,* η*^2^s > 0.31). Finally, we compared the height of the distribution over the target to the height over a “control” color that was equidistant from the first bet but in the opposite direction as the target (Fig. 4b). If the profiles contain no target information, these two heights should be identical. Instead, bet height was significantly greater for target versus control color on guess and target trials (both *p*s < 0.001, *η*^2^s > 0.23) and marginally significant for swap trials *t*(32) = 1.80. *p* = 0.081, *η*^2^ = 0.09 (notably, swap trials had reduced power due to low trial counts).

We find that participants know something about target identity even when their first response would suggest otherwise. This finding suggests that traditional interpretations of guess and swap trials need to be revisited. Guess responses may reflect issues of report or retrieval more than outright failures to encode the item into memory. The rate of true guessing is likely smaller than what single response methods suggest (see “Supplemental Materials”). Swap responses may reflect a momentary misattribution error^41^ or a strategic guess based on a failed retrieval^19^ instead of a failure of binding during memory consolidation.

A possible concern with the setup of Experiments 1 & 2 is that the results do not necessarily result strictly from visual WM. It could be that there is additional information from verbal WM (e.g., participants verbalizing the colors in the array) or in some lingering perceptual trace. We replicated the experiment with modifications to eliminate the contribution of these sources of memory.

### Experiment 3

**Figure 5:**
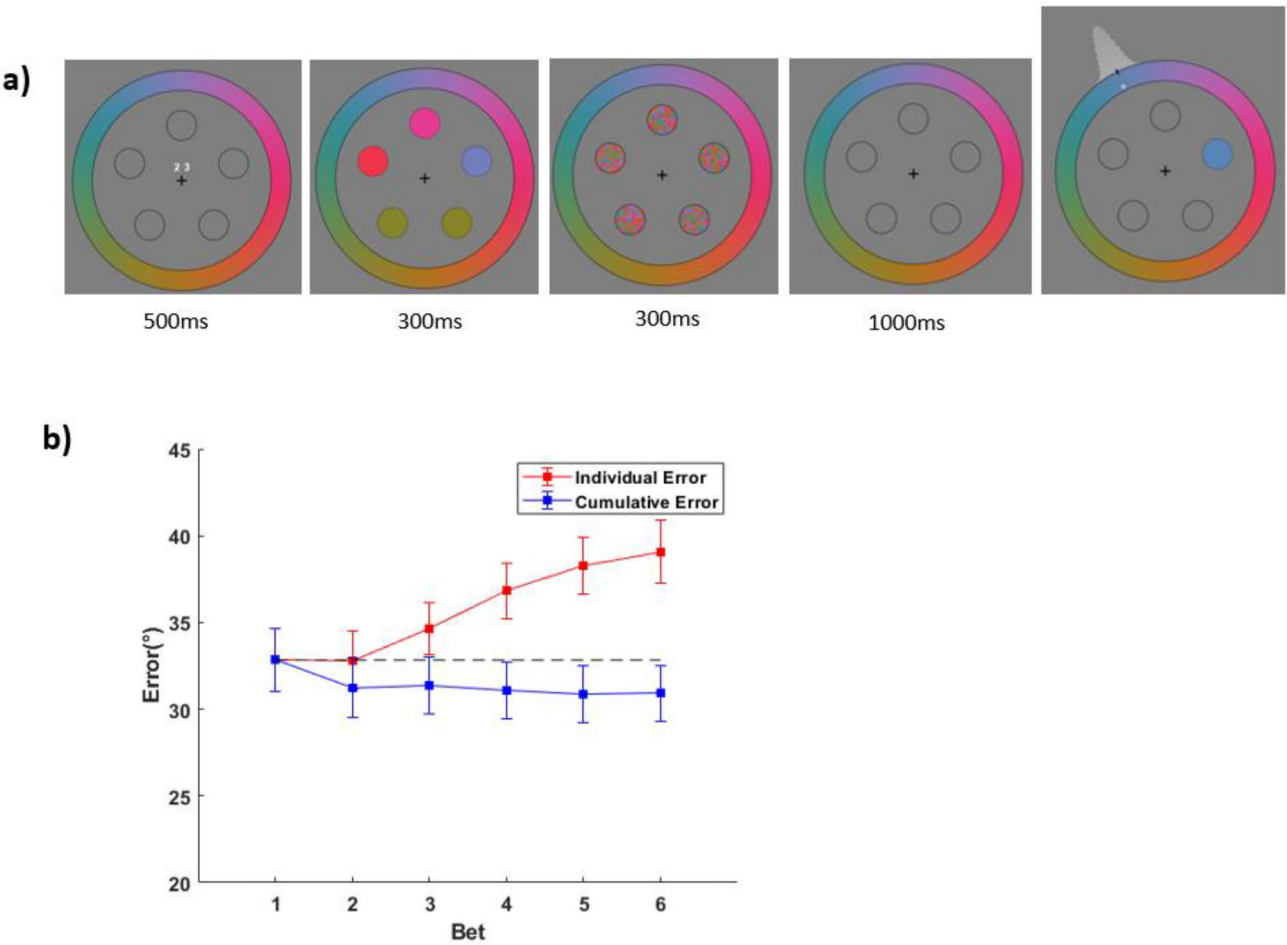
(**a**) Experiment 3 paradigm. As with previous experiments, participants were given 6 bets per trial, only that the bets took place on a color wheel instead of a horizontal bar. The digit probe test occurred after the 6th color bet. (**b**) Individual response errors (red) and cumulative errors (blue) as a function of response order (regardless of digit probe accuracy).

We introduced modifications to Experiment 1 (see Fig. 5). Firstly, participants were explicitly told not to name the colors. To facilitate this, each trial began with a two-digit number (presented for 500 ms) which participants were asked to repeat to themselves/subvocalize^42,43^. Participants were given a probe digit after the color task and asked whether it was one of the two digits shown earlier. Participants were warned that they would not get points for the color task if they were incorrect on the digit task. As a color mask, after the 300 ms of presentation, there was a further 300 ms in which the patches within the five circular markers were replaced with random colors each frame (there are approximately 100 random patches per circle). Lastly, we changed the setup such that the response was done on a color *wheel* instead of a color *bar*. Unlike Experiment 1, the first response did not shift the color space/cause the Gaussian to appear in the middle of the response (bar). This was to demonstrate that the results are generalizable and not dependent on the exact design of the betting task. Bet 1 now behaved exactly like Bets 2–6, aside from the Gaussian component being twice as tall. Each Gaussian component now had a width (standard deviation) of 4° instead of 6°. Again, this was done to ensure that the results are generalizable and that uncertainty profiles drawn are not dependent on specific bet shape components. Otherwise, the score system was the same as Experiment 1 & 2. We ran this experiment on 40 naïve participants (9 female, 29 male, 2 self-identified as non-binary, age range 18–32 [median = 21]). Four sets of data were removed because > 66% of the trials were guesses.

## Results

For the digit probe test, participants scored an average of 76.3% (*SD* = 10.1%). Other trends closely mirrored that of Experiments 1 & 2. Bet spread correlated with median absolute error (mean *r* = 0.301), with 30/36 participants showing this significantly (*p* < 0.05). The shape of the distribution of 1^st^ bet errors across trials closely matched that of the average uncertainty profiles, with 31 of 36 participants having a non-significant *KS*-test (*p* > 0.05), (mean *KS* statistic = 0.041).

Individual bet errors increased across bets, *F*(5,175) = 16.20, *p* < 0.001,* η*^2^ = 0.316, while cumulative bet errors decreased across bets, *F*(5,175) = 3.32, *p* = 0.007,* η*^2^ = 0.087. Importantly, equivalent results were found if the trials where the digit probes were responded to incorrectly were dropped: individual bet errors increased *F*(5,175) = 14.90, *p* < 0.001,* η*^2^ = 0.299, while cumulative bet errors decreased, *F*(5,175) = 2.64, *p* = 0.025,* η*^2^ = 0.070, ruling out the possibility that bet error decreases arise only due to the presence of unattended-to trials (e.g., where the later best might randomly have better guesses than bet 1). In order to test whether our results generalize to a different stimulus space, we modified the task to use a complex shape space.

### Experiment 4

**Figure 6:**
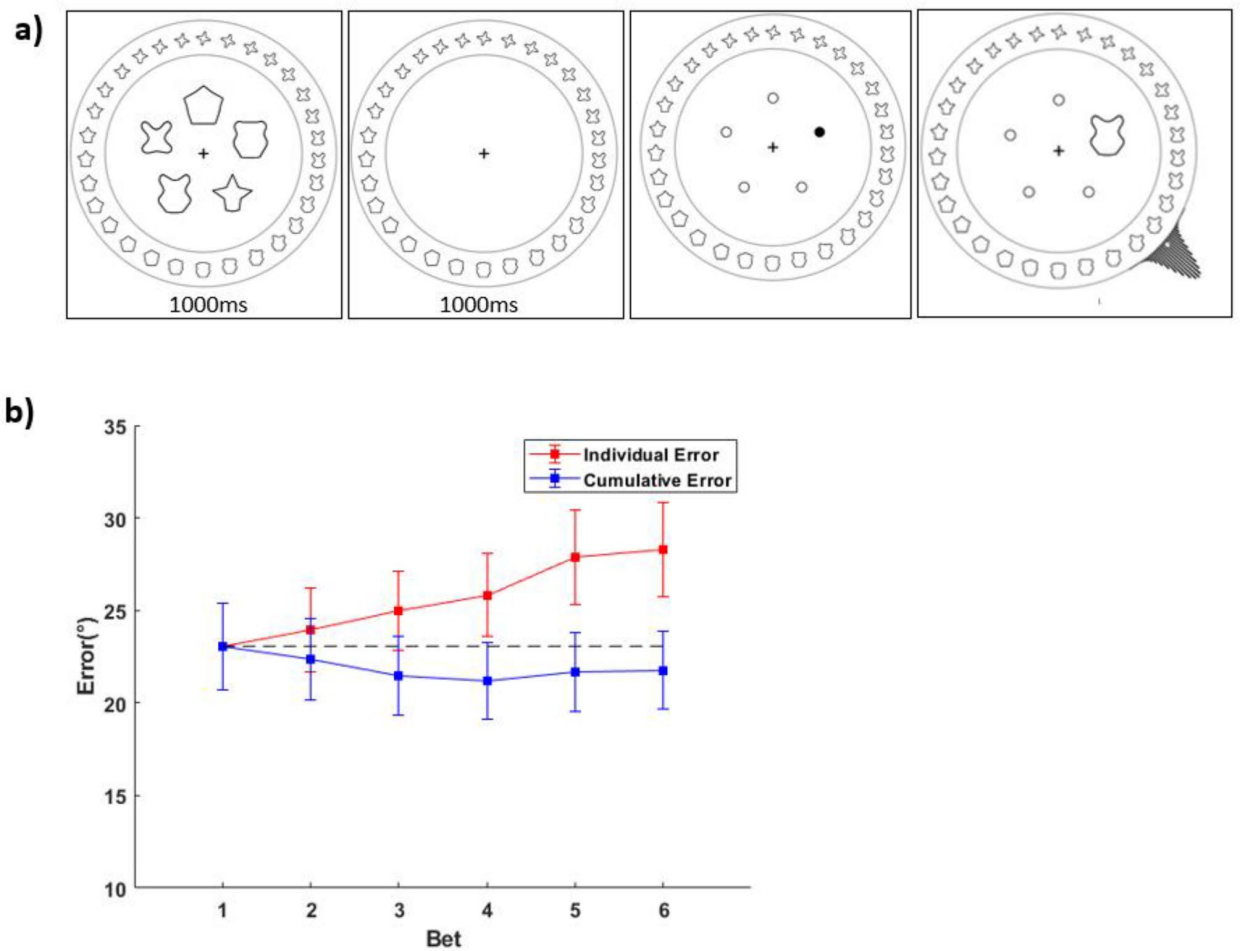
(**a**) Experiment 4 paradigm. Shape stimuli used was adapted from Li et al.^44^. Participants remembered five shapes and later reported an uncertainty profile for the probed shape. As with Experiment 3, six bets were made on a circle. (**b**) Individual response errors (red) and cumulative errors (blue) as a function of response order.

The validated circular shape space^44^, comparable to the color space, consists of 360 shapes. Angular distance along its 2D circle is correlated to visual similarity. As with Experiment 3, the task was performed on a circle (Fig. 6a), with 5 stimuli (shapes, subtending approximately 1.5 times the visual angle of the color circles in Experiments 1–3). Stimulus display duration was 1000 ms. Memory duration was also 1000 ms. To minimize naming of the shapes which might introduce some effect of verbal memory, participants were asked to repeat ‘THE’ across the trial. Forty naïve participants were recruited (15 female, 24 male, 1 self-identified as non-binary, age range 18–31 [median = 22]). Three datasets were removed because of large guess rates > 66%. The feedback and score system was the same as Experiment 3, despite the shift to using shape stimuli.

## Results

Trends for the shape space closely mirrored that of the color space. Bet spread correlated with median absolute error (mean r = 0.379), with 36/37 participants showing this significantly (*p* < 0.05). The shape of the distribution of 1^st^ bet errors across trials closely matched that of the average uncertainty profiles. 30 of 37 participants had a non-significant *KS*-test (*p* > 0.05), (mean *KS stat* = 0.042). Individual bet errors went up across bets, *F*(5, 180) = 14.97, *p* < 0.001,* η*^2^ = 0.294. Critically, cumulative bet errors went down across bets, *F*(5, 180) = 4.54, *p* < 0.001,* η*^2^ = 0.112 (Fig. 6b).

## General discussion

There has been considerable focus on understanding visual WM. Most of this work has focused on categorizing the structural limitations of our WM system—How much information are we able to store and what is the unit of this limit (e.g., objects, features, bits)? However, very little is known about the nature of individual representations and critically, how this information is converted into responses in WM tasks. Researchers have focused on minimizing decision aspects of WM tasks and using analysis techniques that examine the average memory state across many trials. In contrast, an underlying assumption of many models, particularly physiologically plausible models, is that representations consist of patterns of activity that can be effectively described as probability distributions that exist over some feature space^24,25,29^. Unfortunately, there has been a near exclusive focus on evaluating and comparing theoretical models using tasks in which single, discrete responses, sometimes paired with subjective estimates of certainty, are the sole basis of performance. At best, these discrete responses are capturing a representative summary of this probability distribution (e.g., the mode of a Gaussian). However, discrete reports may represent something besides the best estimate of the memory, or the underlying distributions may be sufficiently complex that any method of condensing them into a single response throws away considerable information.

To explore this, and test whether participants have access to a richer representation than simply a point estimate plus a sense of uncertainty, we developed a betting game task where participants were asked to do more than render a single discrete judgment. Instead, participants placed six independent Gaussian bets over a feature space, which were combined cumulatively to form a final response distribution. The trial-specific distributions drawn revealed that participants could access *some* information about their internal representation of the item on a trial-by-trial basis. We found considerably larger response errors for trials in which bets were widely spaced (indicating high uncertainty) than for trials that were closely spaced (indicating low uncertainty). Moreover, the uncertainty profiles drawn by participants on individual trials were comparable to the across trial aggregate distributions, suggesting that participants are tapping into something akin to an internal probability distribution to guide bet placement. This finding mirrors previous studies showing that participants have metaknowledge of memory quality^18,21,32,33^ but goes further in that it suggests memory is richer than simply a sense of uncertainty, since the data suggests that uncertainty profiles are asymmetrical towards the target, indicating participants do not just know how certain they are about their chosen response, but also know which other responses are second or third best (e.g., know more about the actual answer). This is something that the use of symmetrical confidence intervals is unable to capture and is inconsistent with the idea that representations are discrete representations plus a notion of confidence^30^.

Most critically, we find that the uncertainty distributions revealed more than just trial-by-trial memory uncertainty. The average response error for the mean of the uncertainty distribution decreased monotonically from the first to the last bet, even though the average error of each bet increased. This was true even when participants did not know if they were placing one or multiple responses, and even after accounting for motor error at response. This cannot be explained by speed-accuracy tradeoffs, nor by participants supplementing their visual memory with either perceptual or verbal memory. Neither are these findings specific to simple features like color; Memory for complex shapes shows the same pattern of findings.

Because participants’ responses become more accurate when averaging in subsequent responses, this indicates that their initial response did not contain all they knew about the target (e.g., it was not their single best possible guess). This could be because they either do not have full access to internal probability, or at least make sub-optimal discrete responses even when given incentive to be accurate. What might be causing participants to sub-optimally respond given the stimulus encoding? One possibility is that we only have access to discrete samples from internal probability distributions^26,27^. Given that evidence of noisy sampling has been found in many paradigms including decision-making^45^, object recognition^46^, attention^47^, etc., it would not be surprising if our conscious access to the contents of memory at any given instance reflects noisy samples. Indeed, independent samples from an internal probability distribution can explain our findings, including the accumulation of additional information with additional responses (Fig. S1, see Supplemental Materials). Critically, by contrast, strategic bet placement (from a confidence interval, for example) is unable to explain information accrual. The pattern of results can only be explained by a mechanism that produces non-optimal responses (allowing responses to tap into independent pieces of evidence). Whether the mechanism is noisy sampling (or something else akin to that) will require future work. Further, our results do not necessarily entail participants having access to a full probability distribution versus ‘probabilistic-like’ representations that consist of multiple independent samples. However, our results suggest that existing theoretical models are underestimating the amount of information encoded into WM by assuming that the responses gathered in memory tasks are a veridical reflection of memory.

All WM tasks require decision-making. A common strategy has been to minimize these decision-components and to treat the responses as synonymous with representation. Assuming that WM representations are rich and complex, it is impossible to derive a task where the output is a pure reflection of memory. Theoretical or computational models with sufficiently specified mechanisms ought to predict what an internal distribution of uncertainty (on a trial-by-trial basis) might look like. Many models propose rich representations composed of uncertainty distributions over feature space^24,25,48–52^ such as how competing encoded representations might interfere or influence one another in biologically plausible ways^25,39,53^. Research that takes seriously the question of what an individual memory looks like will help to bridge the gap between cognitive models and biologically inspired neural models.

Our work does not completely answer the myriad of questions about the nature of WM, but it does suggest a novel framework that could also be extended to study other aspects of cognition, such as the nature of perceptual representations^54^. Given that our memories are rich and complex, our report methods would do well to embrace this complexity.

### Supplementary Information

Supplementary Information.

## Data Availability

Data collected for this experiment and used in the analyses are available online, as is a demo HTML (https://osf.io/7srv4/).
