# Supplementary material for: Probabilistic and rich individual working memories revealed by a betting game
Source: Sci Rep. 2023 Nov 27;13:20912. doi: 10.1038/s41598-023-48242-x (PMC10684519; doi:10.1038/s41598-023-48242-x)
Supplement: Supplementary file 1 — Supplementary Information. [file 41598_2023_48242_MOESM1_ESM.docx]

Supplementary Materials

To better understand what underlying mechanisms can produce our data we conducted simulations with explicit predictions on how internal uncertainty is converted into multiple bet responses. We simulated a million trials where on each trial the first response was either a random response (24% of trials) or was drawn from a Gaussian (76% of trials) with a standard deviation sampled from a higher order distribution with a mean precision of 30° and a standard deviation in precision of 18°. These parameters were the best fitting variable precision model (Fougnie, Suchow & Alvarez, 2012; Van den Berg et al., 2012; Suchow et al., 2013) on our data set. We consider five possible means of placing bets based on memory representations.

**Model A: Point-estimate stacking**

First, we simulate what would be the trends across bets when participants simply stack subsequent bets on bet 1 (with some small added random noise). Bet 1 is the response error generated by the variable precision model. We make no assumptions about whether participants either only have a discrete representation, or they have a probabilistic representation but are simply reporting the mode of that distribution for every bet. As is evident in Figure S1 (panel a), neither individual errors nor cumulative errors are expected to change across bets under this scenario. This would be true regardless of the parameters used, e.g., changing the guess rate or perceptual SD mean or standard deviation changes the magnitude of error across all bets equally.


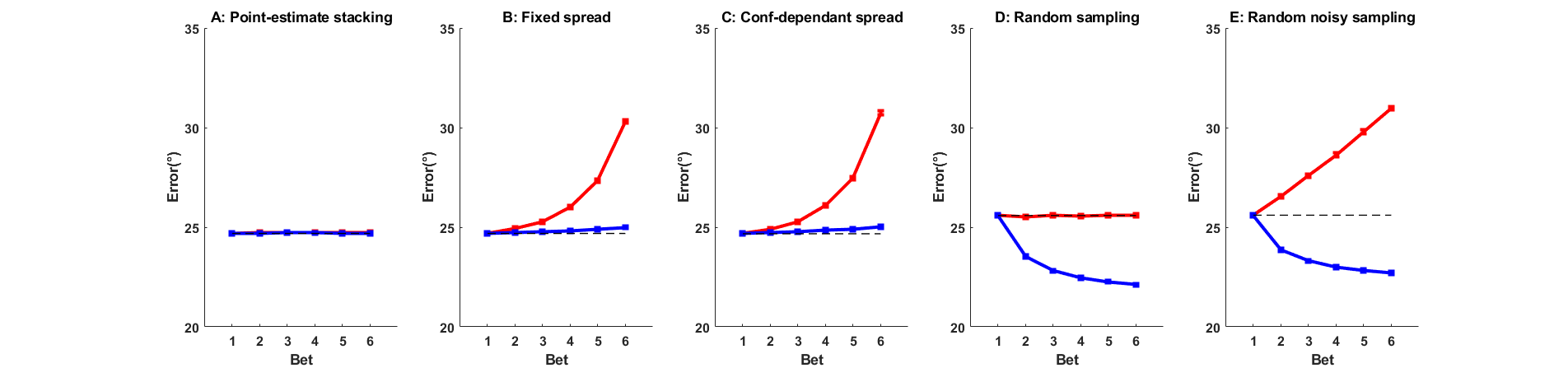


Figure S1. Each panel represents a different mechanism of converting memories into bets. Note that benefits to cumulative error were only seen in the case of sampling.

**Model B: Fixed (confidence-independent) spreading of bets**

A second case (panel b) considers if participants spread bets 2-6 around bet 1. We assume either that participants do not have or do not use a trial-by-trial estimate of uncertainty and spread bets according to the average internal uncertainty. This model starts off like model A, with a mode (bet1) being chosen from the internal uncertainty distribution. Bets 2-6 are then sampled from a Gaussian of this mode, with *SD* of 12° (40% of 30°). We sorted the bets 2-6 in terms of increasing distance from bet 1 (as would be expected if participants fill in their uncertainty profiles outwards from bet 1). Under this scenario, cumulative errors are still unchanging, but note the increase in individual bet error. This *SD* of 12° was chosen just so that individual errors increase to about the same extent as the data., but we wish to highlight there is no bet spread where the cumulative errors decrease across bets (it might in fact, slightly increase).

**Model C: Confidence-dependent spreading of bets**

This model is similar to Model B but assumes that participants have and use an estimate of trial-by-trial uncertainty to influence the amount of spread around bet 1. This could occur either due to participants having a probabilistic representation or a discrete representation plus some sense of confidence (Rahnev, Block, Denison & Jehee, 2021). Here we assumed the same link between *SD* and bet spread, but instead of using the average uncertainty, the true *SD* for that trial provided the basis for spreading bets. This relation was kept linear and at 40% scale. For example, if the perceptual *SD* for a particular trial was 25°, the bet spread around bet 1 for that trial would be 10°, etc. This is somewhat arbitrary, but the mapping of subjective confidence to error can be quite variable across participants (e.g., Jabar & Anderson, 2017). It turns out that the result (panel c) is almost identical to that of a fixed-spread model (panel b). Cumulative errors are still unchanging or slightly increasing) while individual errors increase. The same trends would be observed regardless of how we scale bet spread with perceptual *SD* (e.g., exponential instead of linear). Note that this is not to imply that there is no difference between such models (Model C provides a better explanation of other aspects of our data such as the correlation between bet spread and bet 1 error). However, this demonstrates that knowledge of uncertainty at the individual trial level is not sufficient to explain better performance in cumulative error from additional bets.

**Model D: Sampling**

The previous models have assumed that participants report optimally from the encoded information. However, there are reasons to doubt this assumption. For one, there are multiple lines of evidence that responses may be more akin to random samples from internal probability distributions rather than optimal or representative summaries of this information (e.g., Vul, Hanus & Kanwisher, 2009; Vul, & Pashler, 2008). Similarly, with this model we assume that the stored representation is an uncertainty distribution over color space (we assume a Gaussian) and that bets are samples from this internal distribution. Due to sampling noise, bet 1 will no longer always be the mode (or highest point) of the internal distribution. Similarly, we assume that bets 2-6 will also be samples. For simplicity, the bets are not influenced by previous responses, although one could imagine a strategy of over- or under-correcting bets based on previous responses. An implication of the sampling model is that bets will have more spread than the internal distribution. To simulate a bet 1 error magnitude that is roughly equivalent to the other models, the assumed internal distribution is less uncertain (consistent with our argument that reports overestimate the uncertainty in memory). For this model we modified the mean of standard deviation of the Gaussian to be 85% used in Models A/B/C (although note that the trends remain the same, even if we use the original internal uncertainty spread). This model predicts a benefit to cumulative error, as seen in our data. Of course, this should not be taken to imply that our data can only be explained by internal sampling, but it highlights that (unlike the other models) sampling can produce the same qualitative pattern as our observed data as far as the cumulative benefit is concerned.

**Model E: Sampling with degrading information**

The previous model assumes that the information quality used to place the last sample is as good as that used to place the first sample. This is unlikely, given that response/perceptual interference (e.g., Souza, Rerko & Oberauer, 2016; Suchow, Fougnie & Alvarez, 2017) and time-based decay will result in reduced information quality for later bets. We implemented this by increasing error of later bets by 1° of sampling spread per bet. For example, where bets under Model D for a random trial might be from a Gaussian of mode +9° and standard deviation of 16° for all six bets, this will only be true for bet 1 under Model E, with bet 2 being sampled from a standard deviation of 17° etc. This results in increasing errors for later bets (sloped red line in Figure S1 panel E), although having only a small impact on the benefit of cumulative error. This model is able to replicate the qualitative pattern of data seen in our experiments. Note that the slope of the increase in individual errors can easily be modified by specifying how spread changes across bets differently. We simply gave a linear example to give a basic demonstration.

**Rate of ‘true’ guessing**

In the main paper we highlighted that target information exists even in trials that would traditionally be classified as ‘guesses’ based on a single response. Can we use the 6 bets to predict the true “guessing” rate? Assuming that one were randomly placing bets when guessing, the expected inter-bet distance is 90° (95% CI is [45° 135°]). It turns out that only 12.75% of the trials classified as ‘guesses’ have inter-bet distances in excess of 90°. Further assuming symmetry (that an equal proportion if true guess inter-bet distances fall below 90°), that still only suggests that 1/4 of trials that are classified as guesses from bet 1 are true “guesses” if this strategy is considered the likely thing people do when they have no information. This is another way of pointing out that traditional classification methods lead to an inflated estimate of the proportion of guess trials because participants have more information than is revealed by the first response alone.
